# Magnetic resonance image reconstruction based on image decomposition constrained by total variation and tight frame

**DOI:** 10.1002/acm2.14402

**Published:** 2024-05-23

**Authors:** Guohe Wang, Xi Zhang, Li Guo

**Affiliations:** ^1^ School of Medical Technology Tianjin Medical University Tianjin China

**Keywords:** compressed sensing, image decomposition, magnetic resonance imaging, tight frame, total variation

## Abstract

**Objectives:**

Magnetic resonance imaging (MRI) is a commonly used tool in clinical medicine, but it suffers from the disadvantage of slow imaging speed. To address this, we propose a novel MRI reconstruction algorithm based on image decomposition to realize accurate image reconstruction with undersampled k‐space data.

**Methods:**

In our algorithm, the MR images to be recovered are split into cartoon and texture components utilizing image decomposition theory. Different sparse transform constraints are applied to each component based on their morphological structure characteristics. The total variation transform constraint is used for the smooth cartoon component, while the *L*_0_ norm constraint of tight frame redundant transform is used for the oscillatory texture component. Finally, an alternating iterative minimization approach is adopted to complete the reconstruction.

**Results:**

Numerous numerical experiments are conducted on several MR images and the results consistently show that, compared with the existing classical compressed sensing algorithm, our algorithm significantly improves the peak signal‐to‐noise ratio of the reconstructed images and preserves more image details.

**Conclusions:**

Our algorithm harnesses the sparse characteristics of different image components to reconstruct MR images accurately with highly undersampled data. It can greatly accelerate MRI speed and be extended to other imaging reconstruction fields.

## 1 INTRODUCTION

MRI has been widely used in clinical medicine due to its excellent imaging capabilities of anatomical structures and physiological functions without ionizing radiation.^1^, ^2^ However, because conventional MRI requires sequential acquisition of the entire k‐space data, the speed of MRI is slow, which affects clinical throughput and image quality, especially in dynamic imaging applications. In recent years, compressed sensing (CS) technology has been successfully applied in the field of accelerated MRI. It enables to achieve high‐quality MR image reconstruction with significantly less k‐space sampling data than mandated by Nyquist sampling by utilizing the sparsity of magnetic resonance (MR) images in some transform domains, thus improving MRI speed.^3^, ^4^

The design of sparse transforms of MR images is a crucial factor that significantly impacts the quality of CS reconstruction. Researchers have done a lot of work on constructing sparse transforms of MR images. The total variation (TV) transform used the sparsity of images in the gradient domain to perform MR reconstruction, which preserved the edges of images but was prone to resulting in blocky effects.^5^, ^6^ Orthogonal transforms, such as orthogonal wavelet transforms and curvelet transforms, are widely used in CS because orthogonality is beneficial to theoretical analysis and algorithm design.^7^, ^8^ Compared with orthogonal transforms, tight frame (TF) transforms have achieved better performance in MRI reconstruction.^9^, ^10^, ^11^, ^12^, ^13^ This is because redundant atoms in TF can make the transformed coefficients sparser and better capture different image features than orthogonal dictionaries do. Some representative TFs are patch‐based methods^9^, ^10^, ^11^ and shift‐invariant wavelet frames.^12^, ^13^ Conventional patch‐based methods learned adaptive dictionaries from example image patches to alleviate the drawback of using the fixed basis but might lose important features due to the subdivision of patches.^9^ To make up for this deficiency, a convolutional sparse coding method was proposed to represent the image with a summation of a set of convolutions of the feature maps.^10^, ^11^ With shift‐invariant wavelet frames, a projected fast iterative soft‐thresholding algorithm (pFISTA) was proposed to quickly solve the analysis model and acquire accurate MR reconstruction images.^12^, ^13^ However, the studies mentioned above use a single or mixed set of sparse transforms for the entire image or image patches without accounting for the respective sparsity characteristics of different morphological components of the image, such as smooth parts and texture parts. Although the low‐rank plus sparse model decomposed a full data matrix into two parts, it relied on the spatiotemporal correlations of multiple sequential images, making it suitable for dynamic MRI reconstruction rather than single‐slice MR image reconstruction.^14^, ^15^

In order to better analyze and process images, image decomposition theory has been employed in image denoising,^16^ image fusion,^17^ texture removal,^18^ etc. It decomposes the image into the cartoon and texture components. The cartoon component comprises the smoother structural features of the image, which is usually constrained by the TV transform. The texture component contains the detailed information and noise of the image, which is constrained by the L_1_ norm of sparse transforms.^16^ By applying distinct sparse transform constraints to different components, image decomposition can effectively represent image features.

Capitalizing on the benefits of image decomposition, the contribution of this paper is that we have proposed a novel MRI reconstruction algorithm based on image decomposition constrained by total variation and L_0_ norm of tight frame. It can simultaneously handle both image decomposition into two components and MR image reconstruction. By employing different sparse transforms tailored to each individual component, our method can effectively harness the sparse characteristics of different image components and improve MR image reconstruction performance with highly undersampled data. Specifically, the MR images to be reconstructed were divided into the cartoon and texture components. Since TF transform has superior performance in representing image detail features than orthogonal transforms and L_0_ norm can obtain sparser solutions than L_1_ norm,^19^ we table introduced the L_0_ norm constraint of TF transform to the texture component. For the cartoon component, we adopted the TV transform constraint to represent smooth features. The experimental results under various imaging conditions have demonstrated that our proposed algorithm outperforms two state‐of‐the‐art CS MRI reconstruction methods. Our idea could also be extended to other CS reconstruction fields to improve their performance. In the following sections of the paper, we will use the term ``TV+TFL_0_’’ to denote our proposed MRI reconstruction algorithm, which is based on image decomposition constrained by the TV and L_0_ norm of TF.

The paper is organized as follows: first, the background of CS MRI and image decomposition theory is presented. Then our proposed TV+TFL_0_ algorithm and its solving process are described in the Methods section. In the Results section, numerous experiments are conducted and the experimental results demonstrate the performance of our proposed algorithm. Conclusions and future work are given in the last section.

## 2 BACKGROUND

### 2.1 CS MRI

The undersampled k‐space measurements $y\in C^{M}$ can be briefly described as follows:

(1) $$y=F_{u}x+\eta$$

where $x\in C^{N}$ is the MR image to be reconstructed, $F_{u}\in C^{M\times N}$ (M<N) denotes the undersampled discrete Fourier transform operator, *M* and *N* stand for the number of the *k*‐space measurements and the image pixels, $\eta \in C^{M}$ is the additive noise.

Since M<N, undersampling improves the imaging speed of MR, but it also causes Equation (1) to be underdetermined. CS MRI leverages the sparsity of image transforms to solve this problem. The reconstruction model is expressed as follows^20^:

(2) $$\underset{x}{\arg\min}\lambda {\left\|\Psi x\right\|}_{0}+\frac{1}{2}{\left\|y-F_{u}x\right\|}_{2}^{2}$$

where Ψ denotes the sparse transform, ${∥\cdot ∥}_{0}$ is the L_0_ norm constraint, *λ* serves as the regularization parameter to balance sparsity and data fidelity. Since the L_0_ norm is nonconvex, which is difficult to solve, it is replaced with the L_1_ norm which can be solved efficiently as a convex optimization^20^:

(3) $$\underset{x}{\arg\min}\lambda {\left\|\Psi x\right\|}_{1}+\frac{1}{2}{\left\|y-F_{u}x\right\|}_{2}^{2}$$

### 2.2 Image decomposition

Image decomposition theory states that images can be decomposed into cartoon and texture components to represent different features of the images^17^:

(4) $$x=\;x_{c}+\;\;x_{t}$$

where $\;x_{c}$ and $\;x_{t}$ represent the cartoon component and the texture component of the image x respectively. $\;x_{c}$ is a geometric and smoothly‐varying component, while $\;x_{t}$ is an oscillatory component that contains details and noise. The cartoon‐texture decomposition is commonly obtained through the TV+L_1_ model which consists of a TV constraint and an L_1_ norm constraint of sparse transforms^16^:

(5) $$\underset{\;x_{c},x_{t}}{\arg\min}\frac{1}{2}{\left\|x-\;x_{c}-\;x_{t}\right\|}_{2}^{2}+\lambda_{c}{\left\|\nabla \;x_{c}\right\|}_{1}+\lambda_{t}{\left\|\Psi x_{t}\right\|}_{1}$$

where ${∥\nabla \;x_{c}∥}_{1}$ is the TV constraint, ${∥\Psi x_{t}∥}_{1}$ is the L_1_ norm constraint of a sparse transform Ψ, $\;\lambda_{c}$, and $\lambda_{t}$ are the regularization coefficients of the cartoon component and the texture component, respectively.

## 3 METHODS

### 3.1 Proposed TV+TFL_0_ algorithm

In order to better represent the features of images sparsely and further improve the MR image reconstruction quality, we applied image decomposition theory to CS MRI. Building upon the image decomposition framework in Equation (5), we propose a new MR image reconstruction model named TV+TFL_0_:

(6) $$\begin{array}{ccc} & & \underset{x,x_{c},x_{t}}{\arg\min}\frac{1}{2}{\left\|x-\;x_{c}-\;x_{t}\right\|}_{2}^{2}+\lambda_{c}{\left\|\nabla \;x_{c}\right\|}_{1}\\ & & +\lambda_{t}{\left\|\Psi x_{t}\right\|}_{0}+\frac{\tau }{2}{\left\|y-F_{u}x\right\|}_{2}^{2} \end{array}$$

where the last term represents the MRI data fidelity term, τ is the regularization coefficient for the data fidelity term, and the preceding three terms represent the image decomposition. Despite its greater computational challenge, the L_0_ norm can obtain sparser solutions compared to the L_1_ norm.^19^ Hence the texture component by image decomposition in this paper is constrained using the L_0_ norm. Besides, the sparse transform Ψ adopts a tight frame for better capturing image details, satisfying $\Psi^{*}\Psi =I$, where the superscript * denotes the adjoint operator, and I is the identity matrix.^12^

### 3.2 Solving process

To solve the optimization problem of Equation (6), we employ an alternating iterative minimization approach, wherein one variable is optimized at a time while keeping other variables fixed. Specifically, the solving process is divided into three steps:

Firstly, the cartoon component $x_{c}$ is optimized via Equation (7) with the MR image to be reconstructed x and the texture component $x_{t}$ fixed:

(7) $$\underset{\;x_{c}}{\arg\min}\frac{1}{2}{\left\|x-\;x_{c}-\;x_{t}\right\|}_{2}^{2}+\lambda_{c}{\left\|\nabla \;x_{c}\right\|}_{1}$$

Equation (7) can be solved using the nonlinear conjugate gradient method.^21^

Secondly, we tackle $\;x_{t}$ with x and $x_{c}$ fixed for solving Equation (6) to yield:

(8) $$\underset{\;x_{t}}{\arg\min}\frac{1}{2}{\left\|x-\;x_{c}-\;x_{t}\right\|}_{2}^{2}+\lambda_{t}{\left\|\Psi x_{t}\right\|}_{0}$$

Equation (8) is the L_0_‐norm non‐convex optimization problem, and the iterative hard thresholding algorithm (IHTA) has been demonstrated to be a highly effective solution method.^22^ Besides, Ψ in Equation (8) is a tight frame, and paper^12^ has proposed a fast algorithm called pFISTA to solve the tight frame based L_1_ norm problem. Therefore, here we combine IHTA and pFISTA, denoted as projected fast iterative hard‐thresholding algorithm (pFIHTA), to solve Equation (8):

$$\;x_{t}^{k+1}=\Psi^{*}H_{\gamma \lambda_{t}}\left(\Psi \left({\overset{\wedge }{x_{t}}}^{k}+\gamma \left(x-\;x_{c}-{\overset{\wedge }{x_{t}}}^{k}\right)\right)\right)$$

(9) $$t^{k+1}=\frac{1+\sqrt{1+4{\left(t^{k}\right)}^{2}}}{2}$$

$${\overset{\wedge }{x_{t}}}^{k+1}=x_{t}^{k+1}+\frac{t^{k}-1}{t^{k+1}}\left(x_{t}^{k+1}-x_{t}^{k}\right)$$

where k represents the number of iterations, γ is the step size, and $H_{\gamma \lambda_{t}}\left(q\right)$ denotes the hard thresholding function:

(10) $$H_{\gamma \lambda_{t}}\left(q\right)=\left\{\begin{array}{cc} 0, & \left|q\right|<\gamma \lambda_{t}\\ q, & \left|q\right|\geq \gamma \lambda_{t} \end{array}\right.$$

Thirdly, we get x via the following formulation according to Equation (6) with $x_{c}$ and $x_{t}$ fixed:

(11) $$\underset{x}{\arg\min}\frac{1}{2}{\left\|x-\;x_{c}-x_{t}\right\|}_{2}^{2}+\frac{\tau }{2}{\left\|y-F_{u}x\right\|}_{2}^{2}$$

Equation (11) is the least squares problem and its analytic solution can be obtained as follows:

(12) $$x=F^{-1}\left(\frac{F\left(\;x_{c}+x_{t}+\tau F_{u}^{H}y\right)}{FF^{H}+\tau FF_{u}^{H}F_{u}F^{H}}\right)$$

where F represents the full Fourier transform matrix normalized, the superscript H denotes the Hermitian transpose operation, the matrix $FF_{u}^{H}F_{u}F^{H}$ is a diagonal matrix consisting of ones and zeros, and the ones correspond to the sampled locations in k‐space.^20^

In the first step of the above‐mentioned process, x needs to be assigned an initial value. The initial value is obtained by a tight frame based L_0_ norm optimization problem as shown in Equation (2) when Ψ is a tight frame. Then Equation (2) can also be solved by pFIHTA:

$$\;x^{k+1}=\Psi^{*}H_{\gamma_{1}\lambda }\left(\Psi \left({\overset{\wedge }{x}}^{k}+\gamma_{1}F_{u}^{H}\left(y-F_{u}{\overset{\wedge }{x}}^{k}\right)\right)\right)$$

(13) $$t^{k+1}=\frac{1+\sqrt{1+4{\left(t^{k}\right)}^{2}}}{2}$$

$${\overset{\wedge }{x}}^{k+1}=x^{k+1}+\frac{t^{k}-1}{t^{k+1}}\left(x^{k+1}-x^{k}\right)$$

In summary, the solving steps of the MRI reconstruction model based on TV+TFL_0_ image decomposition proposed in this paper are illustrated in Table 1.

**TABLE 1:** The solving steps of the MRI reconstruction model based on TV+TFL_0_ image decomposition.

Input:y, λ, $\gamma_{1}$, $\lambda_{c}$, $\lambda_{t}$, τ, and γ Output: reconstructed MR image x
(1) Initialization: $x^{0}$ is obtained by Equation (13), $\;x_{t}^{0}=0$ (2) While stop criteria are not met, do: Update $\;x_{c}^{k}$ by using the nonlinear conjugate gradient method to solve Equation (7); Update $\;x_{t}^{k}$ by using pFIHTA to solve Equation (8); Update $x^{k+1}$ by using Equation (12) to solve Equation (11); End

## 4 RESULTS

### 4.1 Experiments

**FIGURE 1:**
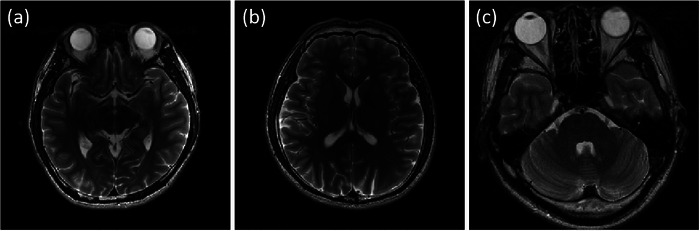
The experimental data of MRI. The parts of figure (a) and (b) are the 7th and the 27th slice of the brain MRI 1. The part (c) is the 31st slice of the brain MRI 2.

The performance of the proposed algorithm has been evaluated by recovering MR images using a variety of sampling schemes at different undersampling factors, with and without noise. Three MR images used in the experiments (the 7th and the 27th slice of the brain MRI 1^23^ and the 31st slice of the brain MRI 2^9^) are shown in Figure 1. The size of all experimental images is 256 × 256. The MR images were normalized to a maximum magnitude of 1.

**FIGURE 2:**
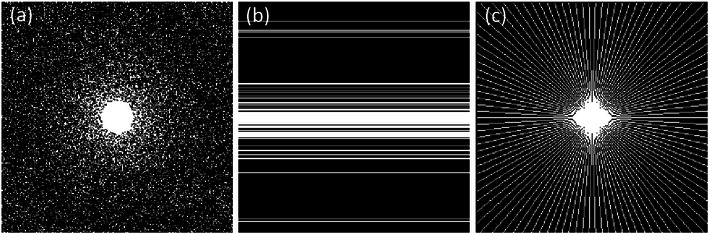
Sampling schemes. (a) 2D variable density random sampling. (b) 1D variable density random sampling. (c) Pseudo radial sampling.

Similar to prior work on CS MRI,^9^, ^20^ the CS data acquisition was simulated by undersampling the 2D discrete Fourier transform of the MR images. The sampling schemes used included 2D variable density random sampling, 1D variable density random sampling and pseudo radial sampling, as depicted in Figure 2. 2D variable density random sampling is usually considered as an ideal case for algorithm verification and can also be used for the 2D phase encodings in 3D imaging in practical applications.^12^, ^21^ In the noisy case, zero‐mean complex white Gaussian noise was added in k‐space with the standard deviation σ= 0.01 or 0.02, which is widely used for noisy model in MRI.^12^, ^24^ Our proposed method TV+TFL_0_ was compared with two state‐of‐the‐art CS MRI algorithms, MCTV‐L_2_^6^ and pFISTA.^12^, ^13^ For MRI reconstruction, MCTV‐L_2_ used a non‐convex isotropic TV constraint and pFISTA adopted a tight frame based L_1_ norm constraint.

In all MR reconstruction experiments, the parameters used in all algorithms were hand‐tuned to achieve the best reconstruction performance in terms of PSNR. For the MCTV‐L_2_ algorithm, the regularization coefficient was set as $\lambda_{TV}$= 0.002, and the augmented Lagrangian parameter ρ= 150. For the pFISTA algorithm, the regularization coefficient was $\lambda_{p}$= 0.001, and the step size was $\gamma_{p}$= 0.1. For the TV+TFL_0_ algorithm proposed in this paper, the parameters were set as λ= 0.03, $\;\gamma_{1}$= 1.3, $\lambda_{c}$= 0.004, $\lambda_{t}$= 0.01, γ= 1.1, τ→+∞ in noiseless experiments and τ = 1000 in noisy experiments. To compare fairly, the tight frames in pFISTA and TV+TFL_0_ were the same, both using shift‐invariant discrete wavelet transform (SIDWT). The shift‐invariant property of SIDWT is beneficial in suppressing artifacts caused by the Gibbs phenomenon in discontinuous regions of the images.^12^

### 4.2 Performance metrics

The quality of the reconstruction results is evaluated by the peak signal‐to‐noise ratio (PSNR) and a high frequency error norm (HFEN). PSNR and HFEN are two commonly used image quality metrics in CS MRI.^9^, ^20^, ^25^

PSNR is defined as^25^:

(14) $$\begin{array}{ccc} & & PSNR=20log_{10}\\ & & \left(\frac{MAX_{I}}{\sqrt{\frac{1}{mn}\sum_{i=1}^{m}\sum_{j=1}^{n}{\left[I\left(i,j\right)-I_{rec}\left(i,j\right)\right]}^{2}}}\right) \end{array}$$

where I is the ground truth image, $MAX_{I}$ denotes the maximum possible value of the image, $I_{rec}$ is the reconstructed image, *m* and n are the number of rows and columns of the image respectively.

HFEN is used to assess the reconstruction quality of edges and fine features. It is calculated as the L_2_ norm of the result obtained by filtering the difference between the reconstructed and ground truth images with a Laplacian of Gaussian filter.^20^

Besides, the error images, which are the absolute value of the difference between the reconstructed images and their corresponding ground truth images in Figure 1, are employed as a visual metric for evaluating the reconstruction quality.^9^

### 4.3 PSNR and HFEN comparison

The PSNR of the reconstruction results of MCTV‐L_2_, pFISTA and TV+TFL_0_ under different undersampling rates and sampling schemes with and without noise are presented in Table 2. It can be observed from Table 2 that the proposed TV+TFL_0_ in this paper greatly improved PSNR compared to the other two algorithms under various imaging conditions for all three test images. Using the imaging condition with the noise standard deviation σ = 0.01, the undersampling rate R = 0.2 and pseudo radial sampling as an example, the PSNR of the 7th slice of the brain MRI reconstructed by MCTV‐L_2_, pFISTA, and TV+TFL_0_ were 29.38, 29.74, and 30.75 dB, respectively (as shown in Table 2). Our method yielded an enhancement in PSNR of more than 1 dB. According to Table 3, TV+TFL_0_ exhibits lower HFEN values than MCTV‐L_2_ and pFISTA in most cases, indicating its superior capability in capturing edges and fine features. Although pFISTA produces slightly higher PSNR than MCTV‐L_2_ in some cases, it generally has the worst HFEN among the three methods. Overall, TV+TFL_0_ outperforms the other two algorithms in terms of PSNR and HFEN.

**TABLE 2:** Comparison of PSNR (dB) of images reconstructed by three algorithms (MCTV‐L_2_/pFISTA/TV+TFL_0_).

				With noise			
Test image	Sampling scheme	Without noise		σ = 0.01		σ = 0.02	
		*R* = 0.2	*R* = 0.3	*R* = 0.2	*R* = 0.3	*R* = 0.2	*R* = 0.3
Figure 1a	2D variable	32.08/32.21/**34.13**	35.93/35.68/**37.65**	31.68/31.78/**33.63**	34.92/34.61/**36.49**	30.70/30.93/**32.26**	32.85/32.97/**34.04**
	1D variable	28.03/27.35/**28.21**	30.51/29.93/**32.45**	27.92/27.22/**28.14**	30.13/29.59/**32.00**	27.56/26.98/**27.71**	29.28/29.04/**30.77**
	Pseudo radial	29.58/29.96/**30.90**	33.76/34.63/**35.47**	29.38/29.74/**30.75**	33.18/33.87/**34.90**	28.84/29.29/**29.84**	31.81/32.63/**33.25**
Figure 1b	2D variable	34.59/34.95/**36.48**	38.58/38.80/**40.24**	33.88/34.07/**35.67**	36.99/36.91/**38.50**	32.43/32.71/**33.73**	34.22/34.64/**35.19**
	1D variable	30.73/30.34/**31.40**	33.32/32.70/**34.46**	30.57/30.17/**31.16**	32.83/32.27/**34.12**	30.05/29.80/**30.67**	31.69/31.46/**32.69**
	Pseudo radial	31.75/32.41/**32.83**	36.02/36.87/**37.27**	31.48/32.05/**32.73**	35.25/35.82/**36.63**	30.72/31.35/**31.72**	33.54/34.19/**34.50**
Figure 1c	2D variable	29.84/29.98/**30.89**	31.61/31.86/**32.73**	29.69/29.86/**30.72**	31.32/31.66/**32.43**	29.27/29.57/**30.18**	30.57/31.15/**31.63**
	1D variable	28.15/27.61/**28.55**	29.76/29.48/**30.77**	28.04/27.55/**28.49**	29.61/29.37/**30.54**	27.74/27.41/**28.12**	29.11/29.10/**29.97**
	Pseudo radial	28.59/28.90/**29.61**	31.25/31.85/**32.43**	28.51/28.83/**29.52**	31.05/31.69/**32.20**	28.24/28.64/**29.06**	30.48/31.26/**31.51**

*Note*: The highest PSNR value is highlighted in bold.

**TABLE 3:** Comparison of HFEN of images reconstructed by three algorithms (MCTV‐L_2_/pFISTA/TV+TFL_0_).

				With noise			
Test image	Sampling scheme	Without noise		σ= 0.01		σ= 0.02	
		*R* = 0.2	*R* = 0.3	*R* = 0.2	*R* = 0.3	*R* = 0.2	*R* = 0.3
Figure 1a	2D variable	0.807/1.002/**0.678**	0.478/0.644/**0.460**	0.872/1.059/**0.732**	0.559/0.729/**0.528**	1.036/1.181/**0.884**	0.771/0.879/**0.706**
	1D variable	**1.638**/1.947/1.665	1.369/1.585/**1.103**	**1.668**/1.981/1.671	1.435/1.637/**1.171**	1.762/2.039/**1.757**	1.581/1.720/**1.327**
	pseudo radial	1.287/1.397/**1.096**	0.560/0.612/**0.462**	1.335/1.445/**1.114**	0.645/0.696/**0.519**	1.458/1.529/**1.247**	0.840/0.835/**0.689**
Figure 1b	2D variable	0.527/0.642/**0.469**	0.318/0.393/**0.295**	0.611/0.732/**0.535**	0.419/0.508**/0.386**	0.793/0.884/**0.720**	0.641/0.673/**0.598**
	1D variable	1.164/1.376/**1.071**	0.885/1.087/**0.806**	1.193/1.404/**1.113**	0.952/1.136/**0.823**	1.294/1.464/**1.183**	1.094/1.222/**0.938**
	pseudo radial	0.895/0.924/**0.771**	0.394/0.433/**0.357**	0.939/0.977/**0.796**	0.475/0.517/**0.422**	1.071/1.078/**0.913**	0.656/0.654/**0.601**
Figure 1c	2D variable	1.355/1.550/**1.250**	1.011/1.164/**0.935**	1.387/1.572/**1.279**	1.045/1.192/**0.974**	1.471/1.625/**1.361**	1.159/1.259/**1.062**
	1D variable	1.830/2.073/**1.772**	1.538/1.747/**1.415**	1.855/2.089/**1.802**	1.563/1.767/**1.457**	1.925/2.121/**1.871**	1.657/1.809/**1.536**
	pseudo radial	1.764/1.876/**1.627**	0.992/1.092/**0.923**	1.778/1.888/**1.625**	1.029/1.117/**0.954**	1.848/1.922/**1.722**	1.133/1.183/**1.038**

*Note*: The lowest HFEN value is highlighted in bold.

**FIGURE 3:**
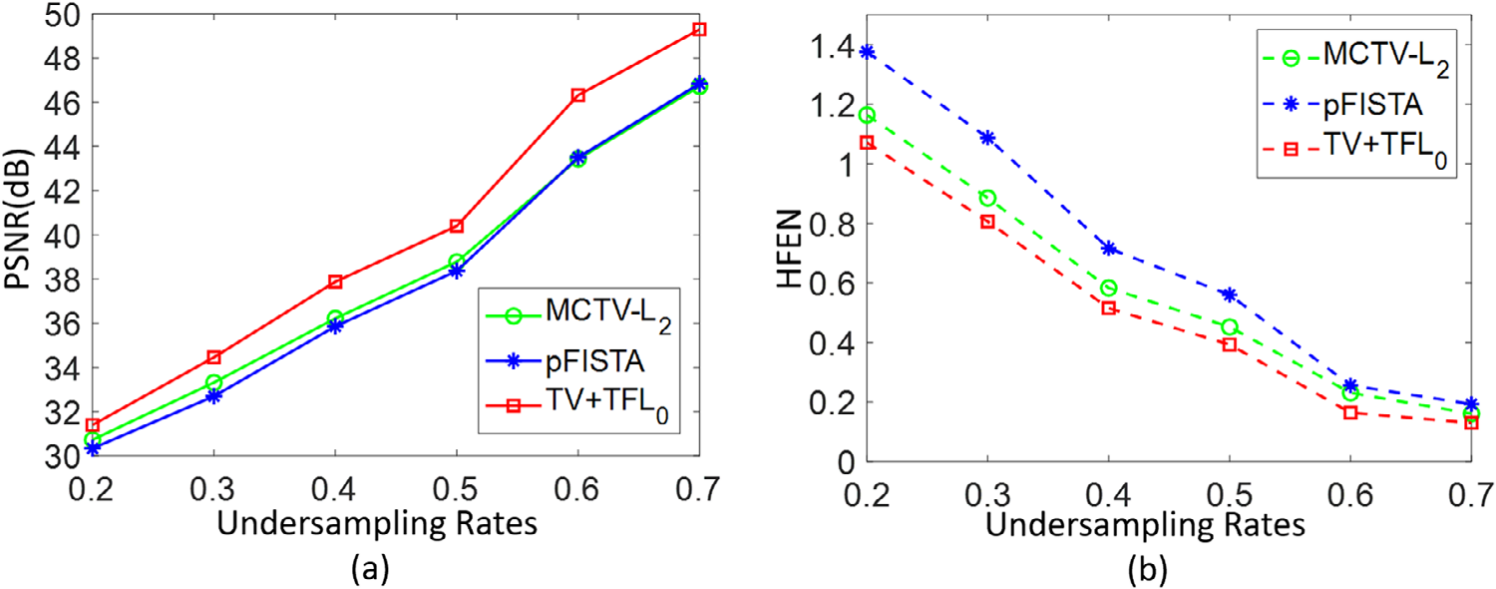
The parts of the figure (a) and (b) are PSNR and HFEN curves of the 27th slice of the brain MRI 1 recovered by MCTV‐L_2_, pFISTA, and TV+TFL_0_ at different undersampling rates.

To further verify the performance of TV+TFL_0_ at different undersampling rates, Figure 3 displays how the PSNR and HFEN values of the 27th slice of the brain MRI 1 reconstructed by the three algorithms change with the undersampling rate from 0.2 to 0.7 under 1D variable density random sampling scheme without noise. From Figure 3 it can be clearly observed that the PSNR and HFEN values of TV+TFL_0_ are better than those of the other two compared algorithms. Hence, we can deduce that TV+TFL_0_ has superior MRI reconstruction performance at various undersampling rates.

### 4.4 Visual inspection comparison

**FIGURE 4:**
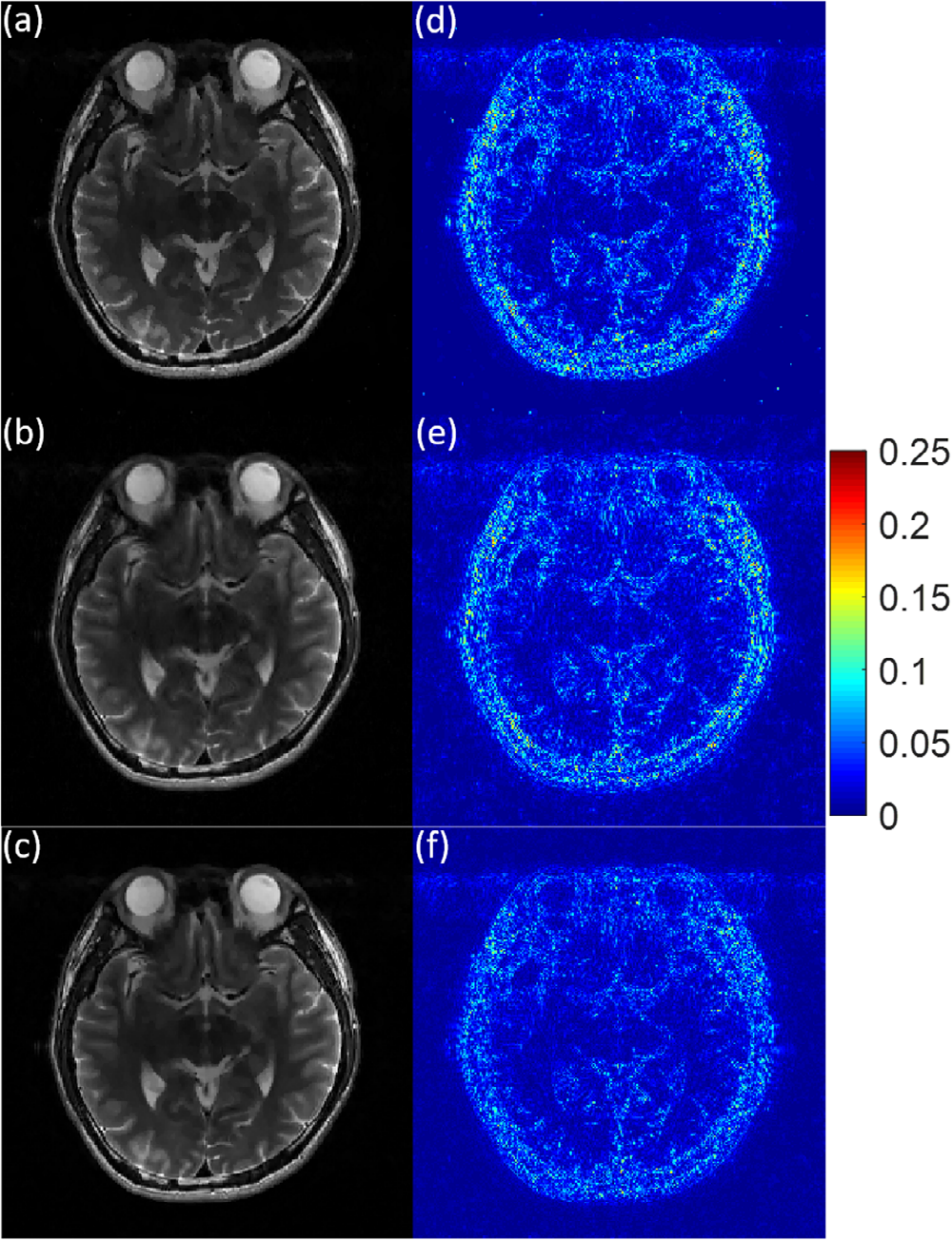
Reconstruction results of the 7th slice of the brain MRI 1. The parts of the figure (a)–(c) are the reconstruction results of MCTV‐L_2_, pFISTA, and TV+TFL_0_. The parts (d)–(f) are corresponding error images of (a)–(c).

**FIGURE 5:**
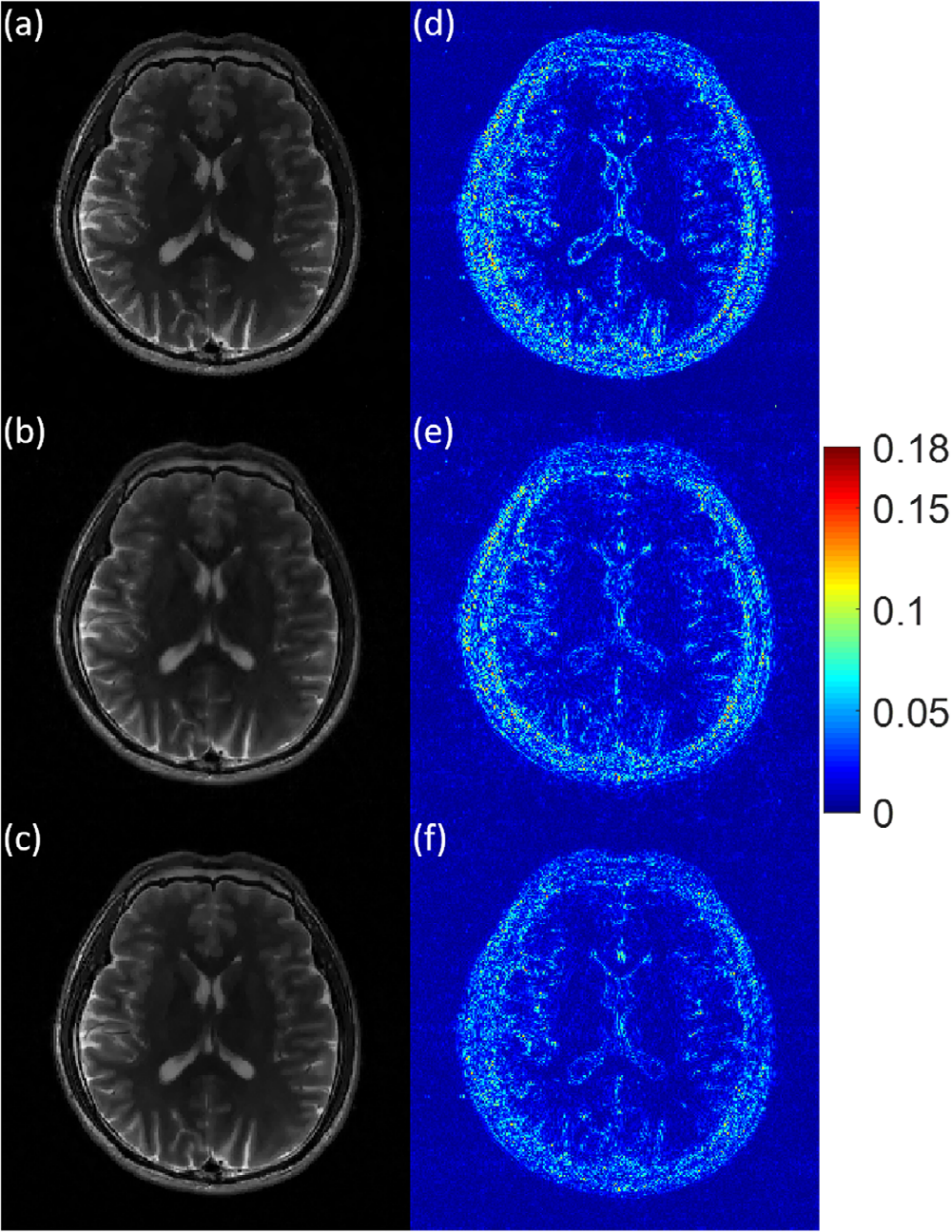
Reconstruction results of the 27th slice of the brain MRI 1. The parts of the figure (a)–(c) are the reconstruction results of MCTV‐L_2_, pFISTA, and TV+TFL_0_. The parts (d)–(f) are corresponding error images of (a)–(c).

**FIGURE 6:**
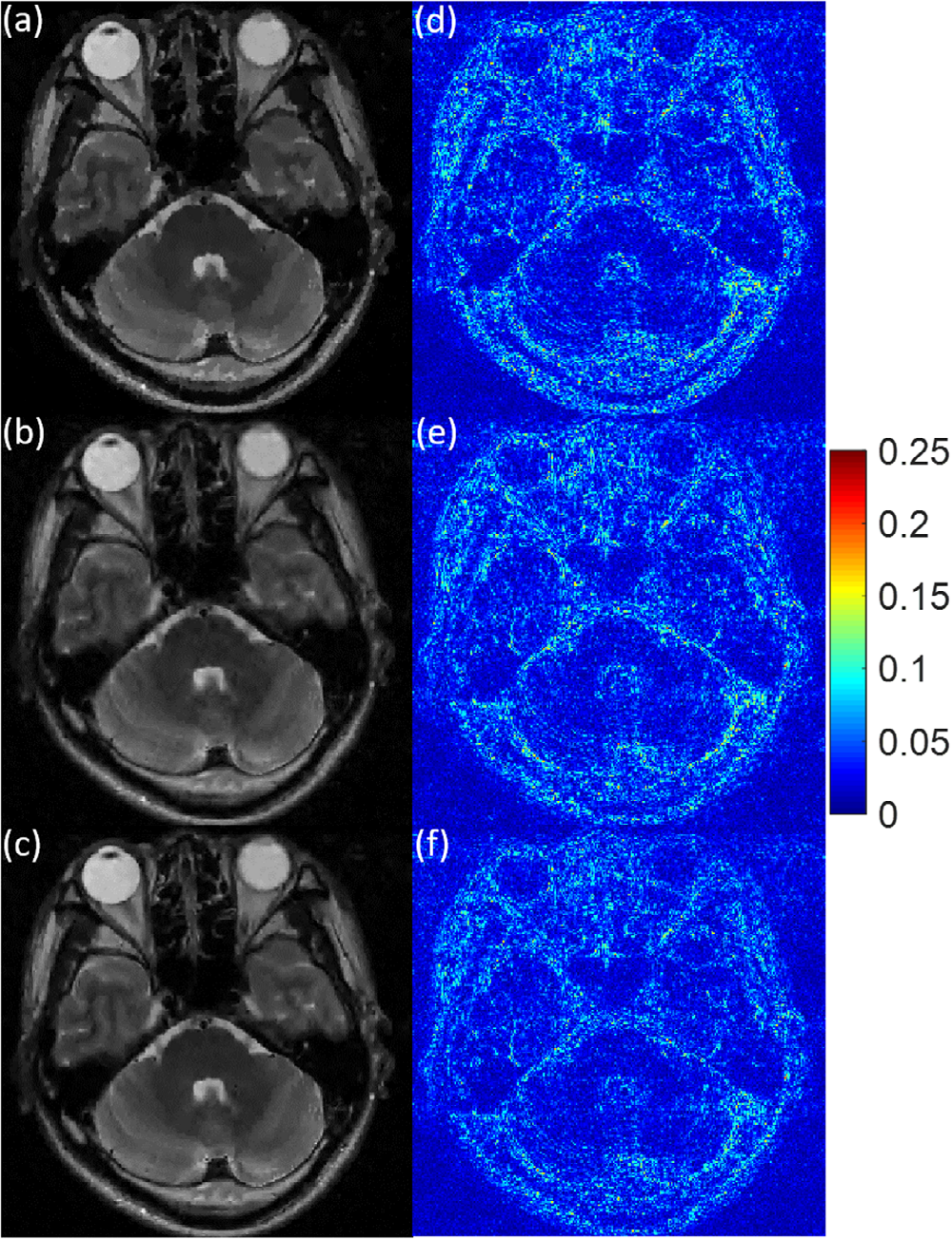
Reconstruction results of the 31st slice of the brain MRI 2. The parts of the figure (a)–(c) are the reconstruction results of MCTV‐L_2_, pFISTA, and TV+TFL_0_. The parts (d)–(f) are corresponding error images of (a)–(c).

To visually assess the image reconstruction quality, the three experimental MR images recovered by MCTV‐L_2_, pFISTA, and TV+TFL_0_ under a 2D variable density random sampling scheme with the undersampling rate R = 0.2 and the noise standard deviation σ = 0.01 are shown in a–c of Figures 4, 5, 6. The corresponding error images are shown in d–f of Figures 4, 5, 6. We can see that the MCTV‐L_2_ algorithm lost many details and produced blocky effects in Figures 4, 5, 6. The error images of the pFISTA algorithm shown in Figures 4, 5, 6 exhibit more visible structures in comparison to those of our TV+TFL_0_ algorithm shown in Figures 4, 5, 6, indicating that pFISTA lost more features. The reason is that MCTV‐L_2_ and pFISTA only exploit the sparsity in the finite difference domain and the SIDWT domain respectively, which is insufficient to capture the complex structures within the images. As shown in Figures 4, 5, 6, in contrast to MCTV‐L_2_ and pFISTA, our TV+TFL_0_ method successfully suppressed more artifacts and retained more detail information by utilizing two more suitable sparse transform constraints based on image decomposition theory. Under other imaging conditions as listed in Table 2, compared with the other two algorithms, TV+TFL_0_ also obtained superior reconstructed images, which is similar to the example shown.

### 4.5 Computation cost comparison

For comparing the computation cost of the three algorithms, we conducted the experiment on reconstructing Figure 1c by using 1D variable density random sampling with the undersampling rate R = 0.2 and the noise standard deviation σ = 0.01. The experiment was implemented in Matlab 2019b on a PC with a 3.10 GHz Intel Core i5 CPU and 8.0 GB memory. The CPU running time of MCTV‐L_2_, pFISTA, and TV+TFL_0_ are 6.35, 64.47, and 79.29 s, respectively. As shown in Tables 2 and 3, although TV+TFL_0_ takes a little longer time than that of MCTV‐L_2_ and pFISTA, TV+TFL_0_ can achieve the best reconstruction performance of Figure 1c.

## 5 CONCLUSION

In this paper, we have developed a novel MRI reconstruction model based on TV+TFL_0_ image decomposition for better sparse representation of MR image features. According to image decomposition theory, we applied distinct sparse transform constraints to the cartoon and texture components according to their respective distinct structural characteristics. For the cartoon component, the TV transform constraint was employed to represent smooth features. For the texture component, the L_0_ norm constraint based on tight frame redundant transform was utilized for capturing detailed features. Compared with MCTV‐L_2_ and pFISTA algorithms, the results of various experiments have demonstrated that our proposed TV+TFL_0_ achieved superior performance for reconstructing MR images by both quantitative and qualitative visual evaluations, such as improved PSNR and HFEN, enhanced detail clarity and reduced artifacts. However, due to the cartoon‐texture image decomposition and their respective different sparse transform constraints, the computational complexity and parameters that need to be tuned increase. In the future, we will study how to speed up sparse transform learning and automatic regularization parameter selection method for the proposed algorithm.

## AUTHOR CONTRIBUTIONS

Guohe Wang: methodology, software, and writing; Xi Zhang: data analyses; Li Guo: conceptualization and supervision. All authors reviewed and edited the manuscript.

## CONFLICT OF INTEREST STATEMENT

The authors declare no conflicts of interest.
